# 

**Published:** 1891-08-29

**Authors:** 


PRICE ONE PENNY.

No. 257, Vol. X.—August 29th, 1891.

[Registered at the General Post Office as a Newspaper for transmission in the United Kingdom and Abroad.]

WITH EXTRA NURSING SUPPLEMENT.

Per Annum, 6s. 6d.; post free.

Monthly Parts, 6d.; post free 8d.

Ditto per Annum, 8s., post free.

### THE HOSPITAL

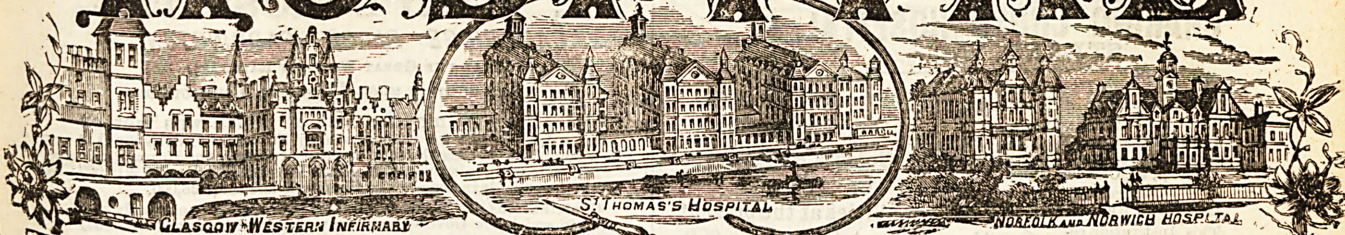

A WEEKLY JOURNAL OF

Science, Medicine, Nursing and Philanthropy.

SATURDAY, AUGUST 29th, 1891.

**Wanted Fair Play for the Voluntary Hospitals** - 253

**Passing Topics.**—A Gift of Days - 254

**Hypnotism** - 255

**The Eastern Hospital Inquiry.**—The Report of the Local Government Board - 256

**Everybody's Page** - 257

**Notes and News** - 258

**General Charities—Scraps and Gleanings** - 259

**The Student of Medicine.**—Syphilis: Its Transmission and Treatment—Appointments—Vacancies—Scholarships and Prizes—Pass Lists

**The Practitioner's Mirror and Retrospect—**

MEDICINE.—Gastrectasis. C—Treatment - 261

SURGERY.—Diseases of the Fingers (*continued*). D—Diseases of the Phalangeal Bones and Joints - 261

THERAPEUTICS.—On the Chemistry and Physiological Action of the Salicyl Compounds - 262

NEW DRUGS, APPLIANCES, AND THINGS MEDICAL.—At the British Medical Association. Section A—Food and Drugs - 263

**Annotations.**—Wife-Beating—The Rating of Hospitals—Dying Daily - 260

**EXTRA SUPPLEMENT—THE NURSING MIRROR.**

**En Passant.**—Rare Courage of a Nurse—Nursing in Workhouses—Kent Nursing Association—The Qualities Requisite in a Nurse—The Hitchin Nursing Institute - cxxv

**Asylum Articles.** VI.—Picturesque Incidents - cxxvi

**Christmas Competitions** - cxxvi

**Nursing in St. Kilda.** By C. G. FURLEY - cxxvii

**The Princess of Wales' Fund for Mrs. Grimwood** cxxviii

**Appointment** - cxxviii

**Wants and Workers** - cxxviii

**The Princess of Wales and the Nurses** - cxxvii

**Everybody's Opinion.**—Drunken Nurses—Wanted Two Hundred and Fifty Pounds—Hurry up, Nurses!—A Matron's Duties in Regard to Nurses' Food - cxxix

**Notes and Queries** - cxxix

**Off Duty.**—On Pleasure Bent - cxxx

**Amusements and Relaxation** - cxxx

